# Machine learning to predict extubation success using the spontaneous breathing trial, objective cough measurement, and diaphragmatic contraction velocity: Secondary analysis of the COBRE-US trial

**DOI:** 10.2478/jccm-2025-0009

**Published:** 2025-01-31

**Authors:** Fabio Varón-Vega, Eduardo Tuta-Quintero, Adriana Maldonado-Franco, Henry Robayo-Amórtegui, Luis F Giraldo-Cadavid, Daniel Botero-Rosas

**Affiliations:** Fundación Neumológica Colombiana, Bogotá, Colombia; Fundación Cardioinfantil Instituto de Cardiología, Bogotá, Colombia; Universidad de La Sabana, Chía, Colombia

**Keywords:** critical illness, airway extubation, ventilator weaning, machine learning

## Abstract

**Introduction:**

Determining the optimal timing for extubation in critically ill patients is essential to prevent complications. Predictive models based on Machine Learning (ML) have proven effective in anticipating weaning success, thereby improving clinical outcomes.

**Aim of the study:**

The study aimed to evaluate the predictive capacity of five ML techniques, both supervised and unsupervised, applied to the spontaneous breathing trial (SBT), objective cough measurement (OCM), and diaphragmatic contraction velocity (DCV) to estimate a favorable outcome of SBT and extubation in critically ill patients.

**Material and Methods:**

A post hoc analysis conducted on the COBRE-US study. The study included ICU patients who underwent evaluation of SBT, OCM, and DCV. Five ML techniques were applied: unsupervised and supervised to the data in both a training group and a test group. The diagnostic performance of each method was determined using accuracy.

**Results:**

In predicting SBT success, all supervised methods displayed the same accuracy in the training group (77.3%) and in the test group (69.6%). In predicting extubation success, decision trees demonstrated the highest diagnostic accuracy, 89.8% for the training group and 95.7% for the test group. The other supervised methods also showed a good diagnostic accuracy: 85.9% for the training group and 93.5% for the test group.

**Conclusions:**

In predictive models using OCM, DCV, and SBT as input variables through five ML techniques, decision trees and artificial neural networks demonstrated the best diagnostic performance. This suggests that these models can effectively classify patients who are likely to succeed in SBT and extubation during the weaning process from mechanical ventilation.

## Introduction

Invasive mechanical ventilation (IMV) involves endotracheal intubation to allow the ventilator to deliver oxygen to the lungs through positive pressure, preventing alveolar collapse during this process [1,2]. Successful weaning (SW) from ventilation is defined as the absence of ventilatory support for at least 48 hours after extubation [3,4]. Weaning failure (WF) occurs when a patient does not pass the SBT and needs reintubation or dies within 48 hours following extubation [4,5]. Approximately 15.6% of intubated patients may develop WF, which is associated with variables such as prolonged mechanical ventilation, advanced age, among others [3,4,5,6].

Comprehensive evaluation and the development of predictive models that consider clinical variables such as SBT, cough strength, acid-base balance, oxygen parameters, diaphragmatic ultrasonographic characteristics, pulmonary compliance, and diaphragmatic function have allowed for the estimation of the risk of weaning and extubation failure [3,4,7]. However, the extensive amount of clinical information can make synthesis and interpretation difficult. Therefore, predictive models based on ML can be used to leverage their ability to detect patterns and analyze large amounts of information [8,9].

Currently, ML is a useful tool in constructing prediction models for SW [8,9,10,11,12,13,14,15,16]. Otaguro et al. [14] analyzed the utility and accuracy of ML to predict WS within the next 72 hours. The three algorithms used in this study showed an area under the receiver operating characteristic curve (AUROC) of 0.950 for LightGBM, 0.946 for XGBoost, and 0.930 for Random Forest. These models utilized variables such as the duration of mechanical ventilation, inspired oxygen fraction, positive end-expiratory pressure, maximum and mean airway pressures, and the Glasgow Coma Scale [14]. However, an ML model must adequately balance clinical characteristics, laboratory tests, and bedside assessments to ensure the model's validity and biological plausibility [8,14,16].

The COBRE-US trial evaluated various tests to determine success in SBT and extubation [7]. An equation derived from cough assessment and diaphragmatic contraction velocity (DCV), with a threshold of ≥ 0.83, showed an accuracy of 76.2%. Conversely, the success of extubation, calculated using a formula that incorporates SBT, OCM, and DCV with a cutoff of ≥ 1.25, demonstrated an accuracy of 91.5% [7]. However, the development of new predictive models or the improvement of existing ones could reduce the risk of reintubation and decrease complications such as morbidity and mortality associated with extubation failure [8,14,15,16].

Statistical models like logistic regressions cannot represent complex and non-linear relationships in clinical data, limiting their accuracy and robustness for predicting future events based solely on stationary variables [9,10]. The use of artificial intelligence (AI) as a viable tool to enhance the predictive capability of clinical variables in critically ill patients requiring IMV could have a positive impact on clinical outcomes [8,14]. Therefore, we believe that applying machine learning techniques to the clinical variables included in logistic regression models, specifically developed to predict success in the spontaneous breathing trial and extubation [7], could improve predictive capability compared to using any of these methods separately [8,16]. Considering the good results obtained with logistic regression predictive models described in the study by Varón-Vega et al. [7], we decided to optimize these results using five AI techniques, both supervised and unsupervised.

## Material and Methods

We conducted a multicenter, observational analysis in adult patients requiring IMV in four ICUs in Bogotá, Colombia. Recruitment took place between February 2019 and November 2021. The primary objective was to apply AI to the results of the study by Varón-Vega et al. [7], using ML techniques to predict success of SBT and extubation.

### Eligibility Criteria

Inclusion criteria for the study were adult patients aged 18 and older who needed IMV for over 48 hours and fulfilled the requirements to begin the weaning process. Patients were required to exhibit a robust cough, show no respiratory secretions, and have resolved the acute phase of the condition that necessitated IMV. They must have a stable cardiac state, without the need for vasopressor support or with minimal doses, an acid-base balance, euthermia, hemoglobin >7 g/dl, a Glasgow Coma Scale score >12, and negative Confusion Assessment Method for the ICU (CAM-ICU). Additionally, the patient must maintain a SaO2 >90% with FiO2 ≤0.4, a PaO2/FiO2 ratio >150 mmHg, and a PEEP ≤8 cm H2O. Patients with acute brain injury, neuro-surgical intervention, pregnant women, and those with neuropsychiatric diseases or diaphragmatic paralysis were excluded from the study.

### Variables

Variables analyzed included sociodemographic variables, the cause of respiratory failure, arterial blood gases before extubation, the mode of ventilation during the weaning process, duration of weaning, days from ICU admission to the start of weaning, and the total length of stay in the ICU. Patients at risk of WF were treated with non-invasive mechanical ventilation (NIMV) or high-flow nasal cannula (HFNC) oxygen therapy.

All patients underwent a 30-minute SBT using a T-piece or pressure support ventilation [6,17]. The criteria for test failure are described in the Appendix 1. The SBT was discontinued in cases of test intolerance [6,7,8,9,10,11,12,13,14,15,16,17].

In the OCM, normal saline (0.9%) was infused via closed suction at the end of inspiration, and peak expiratory flow during the resulting involuntary cough was measured [18]. Ventilatory parameters were set to spontaneous mode without assistance during cough assessment. The objective classification of peak expiratory flow induced by cough is described in the Appendix 2 [18].

A diaphragmatic ultrasound in M-mode was performed to measure DCV (slope, cm/s) from three consecutive normal breaths, with average values used in the analysis [19,20,21]. Assessments were conducted with the patient in a supine position, utilizing either a subcostal or intercostal approach at the midclavicular or anterior axillary lines. The chosen side for measurement depended on technical ease and the clinical judgment of the ultrasound evaluator.

### Analysis Methods

Five ML techniques were used to predict success of SBT and extubation. OCM and DCV were used as predictors for SBT success. On the other hand, OCM, DCV, and successful SBT were used as predictors for extubation success. The study employed five ML techniques, which included two unsupervised methods (hierarchical clustering and k-means clustering) and three supervised methods (support vector machines, decision trees, and neural networks) [22,23]. Each technique underwent training using 10-fold cross-validation. Furthermore, patients were randomly allocated to either the training or test group in a 90/10 ratio. This allocation created a test group that was not involved in training ML techniques [22,23]. This approach enables the assessment of the system's predictive performance on both the training samples and on new samples, mirroring a real-world scenario.

The unsupervised methods were used to let the computer try to find patterns that might reveal interesting associations. Hierarchical clustering groups patients depending on how similar they are, forming pairs and then grouping similar pairs to form a dendrogram [22,23]. On the other hand, k-means clustering groups classify patients by grouping them according to their location in an n-dimensional space. Regarding the supervised methods, the decision tree is like a series of yes or no questions that are easily understood and can be clinically replicated. Support vector machines are a method that involves finding an n-dimensional surface equation that separates the patients into an n-dimensional space [22,23]. And artificial neural networks simulate human brain function by having layers of nodes (neurons) connected and in each node, mathematical operations are applied to the data, leading to a classification probability for each class of patient [22,23].

The classification of patients by each technique, both in the training and test samples, was compared with the actual clinical outcomes. The diagnostic performance of each method was determined using accuracy, sensitivity, specificity, positive predictive value (PPV), and negative predictive value (NPV) [22,23,24].

### Statistical Analysis

Categorical variables are reported as both absolute and relative frequencies [24]. Continuous variables are described using means and standard deviations (SD) or medians and interquartile ranges (IQR), based on their distribution. The Shapiro-Wilk test was used to evaluate the distribution of continuous variables [24].

### Data Analysis and Modeling Software

Data were collected using REDCap software (25). Machine learning models were trained and tested using MATLAB Release 2023a (The MathWorks, Inc., Natick, Massachusetts, USA). Data were analyzed using IBM SPSS software (25, IBM Corp., Chicago, IL, US).

### Ethical considerations

The studies involving human participants were reviewed and approved by the Ethics Committee of the Fundación Neumológica Colombiana (approval number: 201806-23607). Although it is considered minimal risk research, in which no intervention will be carried out, the signature of an informed consent was collected.

## Results

The study included 367 patients, of whom 219 (59.7%) were male. The median age was 61 years, with a range of 18 to 88 years. The mean weight was 70 kg (IQR 60 – 80 kg) and the mean height was 163.6 cm (SD 10 cm). The median BMI was 25.3 kg/m^2^ (IQR 21.7 – 29.1 kg/m^2^). General characteristics of the population are described in Table 1.

**Table 1.** General Characteristics of the Population.

**Variables n (%)**	**Values**
Male n (%)	219 (59,7)
Age, median (Range)	61 (18 – 88)
Weight in kg, median (IQR)	70 (60 – 80)
Height in cm, mean (SD)	163,6 (10)
Body Mass Index (BMI) in kg/m^2^,	
median (IQR)	25,3 (21,7 – 29,1)
Active smoking, n (%)	33 (9)
Alcoholism n (%)	22 (6)

**Comorbidities, n (%)**	
Diabetes Mellitus	113 (30,8)
Hypertension	173 (47,1)
Asthma	8 (2,2)
Pulmonary Fibrosis	6 (1,6)
Chronic Kidney Disease	69 (18,8)
Chronic Liver Disease	17 (4,6)

SD: Standard Deviation IQR: Interquartile Range.

### Etiology of Respiratory Failure

Seventy-five percent (261/367) of the cases were attributed to hypoxemia (PaO2 < 60, usual FiO2), 14.9% (52/367) to ventilatory failure due to shock, followed by 6.6% (23/367) due to hypercapnia (pH < 7.25, elevated CO2) (Table 2). Ninety-four percent (345/367) of the population were admitted to the ICU for medical reasons.

**Table 2.** Etiology of Respiratory Failure and Reason for Admission to Intensive Care

**Variables**	Values
Shock, n(%)	52 (14,9)
Hypercapnia (pH < 7,25, CO2 elevated), n(%)	23 (6,6)
Hypoxemia (PaO2 < 60, usual FiO2), n(%)	261 (75)
Neuromuscular, n(%)	2 (0,6)
Perioperative, n(%)	10 (2,9)

Reason for ICU Admission, n (%)	
Medical	345 (94)
Surgical (post-surgical only)	22 (6)

ICU: Intensive Care Unit.

### Success in Spontaneous Breathing Trial

The unsupervised methods did not find any interesting patterns and did not have a good classification rate. Among the supervised methods, decision trees achieved the highest accuracy in both the training group (77.3%) and test group (69.6%) (Table 3). The other supervised methods, support vector machines and neural networks, demonstrated the same diagnostic accuracy: 77.3% for the training group and 69.6% for the test group.

**Table 3.** Machine Learning Methods for Predicting Success in Spontaneous Breathing Trial

**Model**	**Study Variable**	**Accuracy**	**Sensitivity**	**Specificity**	**PPV**	**NVP**
k-means	SBT^*^ training	64,0	72,6	31,5	79,9	23,5
	SBT ^*^ test	63,0	72,0	35,7	72,7	38,5

Hierarchical Clustering	SBT ^*^ training	52,7	53,3	50,7	80,2	22,4
	SBT ^*^ test	60,9	54,9	64,3	79,2	40,9

Decision Trees	SBT ^*^ training	77,3	99,9	1,1	NI	1,0
	SBT ^*^ test	69,6	99,9	1,0	NI	1,0

Support Vector Machines	SBT ^*^ training	77,3	99,9	1,1	NI	1,0
	SBT ^*^ test	69,6	99,9	1,1	NI	1,0

Neural Networks	SBT ^*^ training	77,3	99,9	1,0	NI	1,0
	SBT ^*^ test	69,6	99,9	1,0	NI	1,0

SBT: Spontaneous Breathing Trial; NI: Not Informed (occurs when division by zero is encountered). PPV: Positive Predictive Value; NPV: Negative Predictive Value.

* Objective Measurement of Cough and Diaphragmatic Contraction Velocity

### Extubation Success

Like before, the unsupervised methods did not find any interesting patterns and did not show good classification rate. In this case, the decision trees also demonstrated the best diagnostic accuracy with 85.9% for the training group and 95.7% for the test group (Table 4). The support vector machines and neural networks both achieved a diagnostic accuracy of 85.9% for the training group and 93.5% for the test group.

**Table 4.** Machine Learning Methods for Predicting Extubation Success

**Model**	**Study Variable**	**Accuracy**	**Sensitivity**	**Specificity**	**PPV**	**NVP**
k-means	SBT^*^ training	63,4	74,4	35,1	74,7	34,7
	SBT ^*^ test	63,0	76,7	37,5	69,8	46,2

Hierarchical Clustering	SBT ^*^ training	66,4	91,6	8,0	69,7	31,4
	SBT ^*^ test	65,2	90,0	18,8	67,5	50

Decision Trees	SBT ^*^ training	89,8	98,3	70,4	94,6	68,7
	SBT ^*^ test	95,7	99,9	87,5	99,9	68,7

Support Vector Machines	SBT ^*^ training	85,9	99,0	56,0	95,9	55
	SBT ^*^ test	93,5	99,9	81,3	99,9	81,3

Neural Networks	SBT ^*^ training	85,9	99,0	56,0	95,9	55
	SBT ^*^ test	93,5	99,9	81,3	99,9	81,3

SBT: Spontaneous Breathing Trial; PPV: Positive Predictive Value; NPV: Negative Predictive Value.

* Objective Measurement of Cough, Diaphragmatic Contraction Velocity, and Spontaneous Breathing Trial

## Discussion

This study investigated the diagnostic performance of ML in predicting the success of the SBT and successful extubation, based on OMC, DCV, and SBT. Among the various combinations evaluated, decision trees and artificial neural networks demonstrated the best diagnostic performance. This finding suggests that these features have the potential to accurately classify patients who are likely to succeed in the SBT and extubation during the weaning process.

Our results indicate the potential for an AI-based decision support system that complements and improves upon simple predictive models constructed with routinely used clinical variables in patients requiring extubation in the ICU [7,18]. We believe these findings could encourage the adoption of ML in critical care, given that physicians often underestimate and, consequently, distrust the potential clinical benefits of supervised techniques such as decision trees and artificial neural networks [8,9,15,26].

Otaguro et al. [14] investigated the utility and accuracy of ML using three algorithms (Random Forest, XGBoost, and LightGBM) to predict extubation success, employing 57 clinical variables that included patient demographics, vital signs, laboratory results, and ventilator information. The results showed an accuracy of 0.897 for Random Forest, 0.910 for XGBoost, and 0.927 for LightGBM. In contrast, our study relies on bedside tests and ultrasonographic measurements validated in epidemiological studies to predict extubation success, thus enhancing predictive capacity by combining statistical and AI techniques [26,27,28]. Moreover, using a reduced number of input variables can improve real-time utility in the ICU and facilitate better physician understanding of the pre-prediction analysis process [26,27,28].

Our study aimed to develop a predictive model utilizing the SBT, routinely performed during the weaning process, due to its critical role as an indicator of weaning outcomes in ICU patients [7,29,30,31]. Although AI can analyze large datasets and uncover patterns that might elude the medical team and conventional statistical methods like logistic regressions, the reliability of input data such as the SBT and its ability to accurately predict extubation success were crucial for developing a more precise model [29,31].

To explore a wide range of possibilities, five machine learning techniques were implemented [32,33]. Initially, clustering techniques were used to analyze the behavior of our variables; however, their diagnostic performance did not reach the level of supervised methods. Among the supervised techniques, a simple decision tree was tested due to its ease of understanding and application in clinical practice [28,32,33]. Support vector machines, which can be very heavy computationally, were employed for their usual effectiveness in separating data in multidimensional spaces. Finally, despite being the most complex and challenging to interpret, artificial neural networks are the most cited in the available literature and proved to be the most effective in most health related issues [27,28,32,33]. In our case, the decision trees were the technique that obtained the highest classification performance.

### Limitations and Strengths

The observational nature of our study exposes it to the risk of confounding factors. Although our goal was to develop a parsimonious predictive model, we acknowledge the importance of considering additional clinical variables, such as laboratory tests, risk scores, ventilation time, and ventilator settings, to more robustly and clinically substantiate predictions for patients undergoing weaning in ICU, ensuring a more accurate diagnosis by intensivists. Furthermore, dimensionality reduction through principal component analysis could be considered. A limitation of this study is the potential influence of confounding biases on the findings, which should be interpreted within the context of a prospective, multicenter observational study, emphasizing the need for external validation of the results.

This study's strengths are attributed to its prospective design and multicenter approach, derived from the study conducted by Varón-Vega et al. [7]. This post hoc analysis incorporates diaphragmatic ultrasound, OCM, and bedside tests into its predictive models, which have been validated with good reproducibility and predictive capability when used individually or in combination. Additionally, intensive care staff were trained to perform transthoracic ultrasound and tests. However, further research is needed to explore the potential of different models that include other clinical variables.

## Conclusion

Among the predictive models that used MOT, VCD, and the SBT as input variables through five machine learning techniques, decision trees and artificial neural networks demonstrated the best diagnostic performance. These models excelled in accurately classifying patients regarding success in the SBT and extubation during the weaning process.

## Abbreviations

AI: Artificial Intelligence
ARF: Acute Respiratory Failure
AUROC: Area Under the Receiver Operating Characteristic Curve
DCV: Diaphragmatic Contraction Velocity
EI: Endotracheal Intubation
HFNC: High-Flow Nasal Cannula
ICU: Intensive Care Unit
IMV: Invasive Mechanical Ventilation
IQR: Interquartile Ranges
ML: Machine Learning
NIMV: Non-Invasive Mechanical Ventilation
NPV: Negative Predictive Value
OCM: Objective Cough Measurement
PEEP: Positive End-Expiratory Pressure
PPV: Positive Predictive Value
SD: Standard Deviations
SBT: Spontaneous Breathing Trial
SW: Successful Weaning
WF: Weaning Failure Abbreviations:

## Appendix 1. Criteria for Failure of Spontaneous Breathing Test

Failure of the spontaneous breathing test was defined as the presence of at least one of the following criteria:

1. Arterial partial pressure of oxygen (PaO2) ≤ 60 mmHg or arterial oxygen saturation (SpO2) ≤ 90% with inspired oxygen fraction (FiO2) ≥ 0.50
2. PaCO2 > 50 mmHg or an increase of > 8 mmHg from the baseline value
3. pH < 7.32 or a decrease of > 0.7 units
4. Respiratory rate ≥ 35/min or an increase of ≥ 50% from the baseline value
5. Heart rate ≥ 140 bpm or an increase of ≥ 20% from the baseline value
6. Systolic blood pressure > 180 mmHg or an increase of ≥ 20% or systolic blood pressure <90 mmHg
7. New-onset cardiac arrhythmias
8. Abrupt change in mental status
9. Presence of two or more signs of respiratory distress:
  ○ Tachycardia
  ○ Bradycardia
  ○ Increased respiratory effort
  ○ Use of accessory muscles
  ○ Facial signs of distress
  ○ Diaphoresis
  ○ Cyanosis
  ○ Dyspnea

## Appendic 2. Objective evaluation of cough.

**Table:**

Qualification	Description
0	No cough
1	Audible movement of air through the endotracheal tube, but no audible cough
2	Strong cough with movement of secretions into the endotracheal tube
3	Strong cough with movement of secretions out (expulsion) of the endotracheal tube
